# Ultrasound-Guided Pulse-Dose Radiofrequency: Treatment of Neuropathic Pain after Brachial Plexus Lesion and Arm Revascularization

**DOI:** 10.1155/2014/429618

**Published:** 2014-11-26

**Authors:** Ernesta Magistroni, Davide Ciclamini, Bernardino Panero, Valter Verna

**Affiliations:** ^1^Physical Medicine and Rehabilitation Department, Trauma Center, CTO Hospital, Via Zuretti 29, 10100 Torino, Italy; ^2^Microsurgery Unit, Trauma Center, CTO Hospital, Via Zuretti 29, 10100 Torino, Italy; ^3^Department of Imaging, Trauma Center, CTO Hospital, Via Zuretti 29, 10100 Torino, Italy

## Abstract

Neuropathic pain following brachial plexus injury is a severe sequela that is difficult to treat. Pulsed radiofrequency (PRF) has been proved to reduce neuropathic pain after nerve injury, even though the underlying mechanism remains unclear. This case report describes the use of ultrasound-guided PRF to reduce neuropathic pain in a double-level upper extremity nerve injury. A 25-year-old man who sustained a complete left brachial plexus injury with cervical root avulsion came to our attention. Since 2007 the patient has suffered from neuropathic pain (NP) involving the ulnar side of the forearm, the proximal third of the forearm, and the thumb. No pain relief was obtained by means of surgery, rehabilitation, and medications. Ultrasound-guided PRF was performed on the ulnar nerve at the elbow level. The median nerve received a PRF treatment at wrist level. After the treatment, the patient reported a consistent reduction of pain in his hand. We measured a 70% reduction of pain on the VAS scale. PRF treatment allowed our patient to return to work after a period of absence enforced by severe pain. This case showed that PRF is a useful tool when pharmacological therapy is inadequate for pain control in posttraumatic neuropathic pain.

## 1. Introduction

Pulsed radiofrequency (PRF) was developed with the goal of providing reduction in pain through the use of electrical fields in the absence of neural injury [1]. Its first application dates back to 1996. The first report of its use, in the dorsal root ganglion, appeared in* The Clinical Journal of Pain* in 1995 [2]. Prospective trials on the use of PRF have shown a beneficial effect on pain reduction in a variety of chronic pain conditions [3–6]. The debate on the mechanisms underlying the effect of PRF continues in the literature [7, 8]. Rat cervical dorsal root ganglia were exposed to PRF, showing an increase in c-Fos immunoreactivity in the dorsal horn up to 1 week after treatment [9]. The potential long-term effect on the targeted nerve is a matter of concern. In vitro and in vivo studies have failed to demonstrate local tissue damage with the application of PRF [10, 11]. Hamann et al. [12] concluded that PRF has a biological effect that is unlikely to be related to overt thermal damage and that it targets small-diameter C and A-delta nociceptive fibers. Some recent laboratory studies (in rats) suggest that pulsed radiofrequency may modulate pain regulatory gene expression along the nociceptive pathway. According to laboratory data, PRF therapy could influence the reversal of molecular effects of hypersensitivity developed from a peripheral nerve injury [13].

PRF is a technique designed to give long-term pain relief, consisting of short bursts of PRF delivered to a target nerve to produce effects on signal nerve transduction. A 50 kHz current is usually delivered in 20 ms pulses at a frequency of 2 Hz for a period of 120 s. The procedure may be ultrasound guided, fluoroscopy guided, or computed tomography guided, depending on the site to be treated. PRF has also previously been used in other fields such as radicular pain, face pain, postsurgical pain, facet arthropathy, myofascial pain syndrome, sacroiliac pain, and groin pain. Ultrasound guidance continues to be useful in regional anesthetic blocks [14–16]. Reports have confirmed its use in the localization of nerves for the treatment of chronic pain conditions [17, 18] and for the positioning of PRF probes in patients with carpal tunnel syndrome [19].

This case report describes the use of ultrasound-guided PRF to reduce neuropathic pain (NP) in a double-level nerve injury of the upper extremity.

## 2. Case Report

### 2.1. Surgical Course

In May 2006, a 25-year-old man, who had sustained a complete left brachial plexus injury with cervical root avulsion and subclavian artery lesion in a motorcycle accident, came to our attention. An exposed distal humeral shaft fracture at the level of the elbow with median and ulnar nerve transection was also identified.

The patient was treated in an emergency department with humeral fracture stabilization (Synthes locking compression plate; Synthes, West Chester, PA, USA) and revascularization of the left arm. In July 2006 he underwent surgery of the brachial plexus lesion, with nerve transfers of the distal spinal accessory to axillary nerve and the phrenic to musculocutaneous nerve. Unfortunately he did not show any sign of recovery of the motor function in the left upper limb, and in August 2008 a functional transfer of a gracilis free flap to brachial biceps and a wrist arthrodesis were performed. Over the last 6 years the patient has undergone surgery 3 times and has followed a rehabilitation program.

### 2.2. Rehabilitation Course

From the outset, the patient has been treated by searching for symmetry in orthostatic posture. The Global Postural Rehabilitation technique has been used thus far in the course of rehabilitation. Three times per week the patient sustains passive mobilization of each upper limb joint to maintain articular suppleness and prevent stiffness. During the session, the physiotherapist also administers sensitive stimulation to maintain afferent channels.

At all times the patient wears splints to place the distal extremity in an intrinsic position for correct articular and muscle length. The whole upper limb is supported by a sling. Postural control, splinting, mobilization, and sensibility rehabilitation have all been included in the rehabilitation program.

### 2.3. Pain History

Since 2007 the patient has suffered from neuropathic pain (NP) involving the ulnar side of the forearm, the proximal third of the forearm, and the thumb in both radial (dorsal) and median (volar) regions.

From the outset until December 2010, NP was successfully controlled with pregabalin, 600 mg/day, and duloxetine, 75 mg/day. On rare occasions severe pain was controlled with oxycodone and paracetamol, but with adverse side effects. Ultimately satisfactory pain relief was obtained with morphine, 60 mg/day.

In January 2011, the NP gradually worsened and became localized in the ulnar side of the hand and the volar thumb. Because of the severe NP, the patient was unable to wear a splint on his forearm and hand. He had continuous pain ranging from 3 to 9 on the Visual Analog Scale (VAS). The pain worsened when the patient was tired and after a busy and active day and was relieved by rest. Night rest was disturbed. As a result of pain worsening, we decided to perform PRF treatment.

## 3. Materials and Methods

The patient was informed that data concerning the case would be submitted for publication, for which he gave his free consent. Before applying the method, the patient underwent EMG investigation (every 6 months since the beginning of the rehabilitation program). EMG showed, for ulnar nerve, a complete absence of MAP (Motor Action Potential) with presence of moderate SAP (Sensitive Action Potential). The median nerve showed complete absence of MAP and SAP.

The PRF procedure was guided by ultrasound using the Esaote (Genoa, Italy) MyLab 30 Gold system with an LA523 probe and a frequency of 7.5 to 12 MHz. The PRF treatment was performed with the NT1100 NeuroTherm (Wilmington, MA, USA) system with a disposable pulse-dose kit.

The needle length was 50 mm (TSS.J050.DTC). A total of 1200 doses (1 dose = 200 mA, 20 ms, 2 PPS, with maximum temperature 42°C) was delivered.

In February 2011 we carried out a diagnostic ulnar and median nerve block with ropivacaine 7.5%. Both tests were positive, with complete pain relief lasting from 6 to 12 hours.

Subsequently a total of 5 ultrasound-guided PRF were performed on the ulnar nerve at the elbow level, with a mean period of pain relief after each treatment of about 4 months. The median nerve received a first PRF treatment at wrist level, resulting in complete pain relief for 6 months.

## 4. Results

After the last treatment at ulnar nerve level, the patient reported a consistent reduction of pain in his hand. We measured a 70% reduction of pain on the VAS scale. The time frame between the PRF treatment and the pain relief was 24–36 hours. The patient showed no side effects related to the PRF treatment.

## 5. Discussion

Brachial plexus injury, frequently observed in young people, is a disability that affects daily work activities. This case showed that PRF is a useful tool when pharmacological therapy is inadequate for pain control. PRF treatment allowed our patient to return to work after a period of absence enforced by severe pain. Pain relief onset was fast, at about 24–36 hours after the treatment. A multidisciplinary approach involving the orthopedist, radiologist, physiatrist, physiotherapist, and algologist is beneficial in achieving the best results. However, a longer follow-up will be needed to evaluate the long-term effectiveness of pain relief. Thus far, this paper seems to be the first published paper addressing the treatment of chronic posttraumatic neuropathic pain with the use of PRF.

Apart from laboratory studies on damaged sciatic nerve injury in rat models, no clear evidence around the mechanism of action of PRF actually exists, both in the chronic pain field and in the chronic posttraumatic neuropathic pain field. As such, a speculation about possible differences in terms of mechanism of action and efficacy with respect to chronic nonposttraumatic pain seems to be quite unsupported by any evidence.

## 6. Conclusion

According to published data, follow-up data suggest that the application of short bursts of radiofrequency energy to the nervous tissue may result in intermediate to long-term pain relief. This procedure does not usually affect motor and sensory recovery, even though a minimal risk of nerve damage has been described [6].

A PRF procedure should be considered as an alternative treatment for all posttraumatic nerve injuries that are complicated by NP. PRF is safe in locations where conventional radiofrequency is potentially hazardous. It may also be applied in more than 1 region without complications.

PRF is useful for the control of NP during mobilization and sensitivity rehabilitation after surgical repair of nerve injury and is an alternative to opioid therapy that potentially jeopardizes the daily lives of young active patients.
